# Breast cancer with biomarker reversal during the course of treatment: a case report

**DOI:** 10.1093/jscr/rjae432

**Published:** 2024-06-28

**Authors:** Ryusei Yoshino, Masaki Nakatsubo, Nanami Ujiie, Masahiro Kitada

**Affiliations:** Department of Thoracic Surgery and Breast Surgery, Asahikawa Medical University Hospital, 2-1-1-1 Midorigaoka Higashi, Asahikawa, Hokkaido 078-8510, Japan; Department of Thoracic Surgery and Breast Surgery, Asahikawa Medical University Hospital, 2-1-1-1 Midorigaoka Higashi, Asahikawa, Hokkaido 078-8510, Japan; Department of Thoracic Surgery and Breast Surgery, Asahikawa Medical University Hospital, 2-1-1-1 Midorigaoka Higashi, Asahikawa, Hokkaido 078-8510, Japan; Department of Thoracic Surgery and Breast Surgery, Asahikawa Medical University Hospital, 2-1-1-1 Midorigaoka Higashi, Asahikawa, Hokkaido 078-8510, Japan

**Keywords:** breast cancer, metastasis, recurrence, biomarker, re-biopsy

## Abstract

During breast cancer recurrence, drug therapy is planned based on the biological characteristics of the primary tumor. However, the mechanisms underlying these changes have not yet been clarified. A 59-year-old woman underwent breast cancer surgery 23 years previously and received postoperative hormone therapy for 2 years. She had abdominal distention and ascites effusion and was diagnosed with carcinomatous peritonitis due to luminal-type breast cancer after ascites puncture. She received up to the fourth line of treatment. Subsequently, pleural effusion was observed and human epidermal growth factor receptor 2 type breast cancer was diagnosed because of pleurodesis. This case suggests that the cell block diagnostic method based on thoracic and ascites fluid cytology is useful not only for confirming the primary tumor but also for diagnosing the biological characteristics of breast cancer. In the treatment of breast cancer recurrence, it is important to plan the treatment, including aggressive re-biopsy of metastases.

## Introduction

On recurrence of breast cancer, drug therapy is often planned based on the biological characteristics of the primary tumor. We have treated cases in which changes in characteristics are observed in the recurrent tumor. However, the mechanism underlying these changes has not yet been clarified [1].

## Case presentation

**Figure 1:**
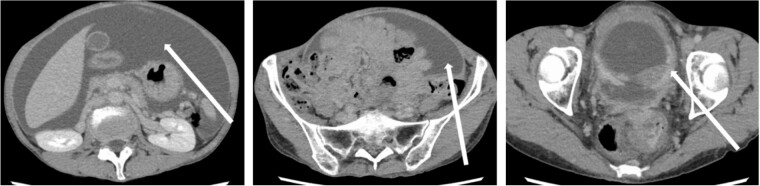
CT findings. In addition to significant ascites accumulation, there was a partial bowel obstruction-like finding.

**Figure 2:**
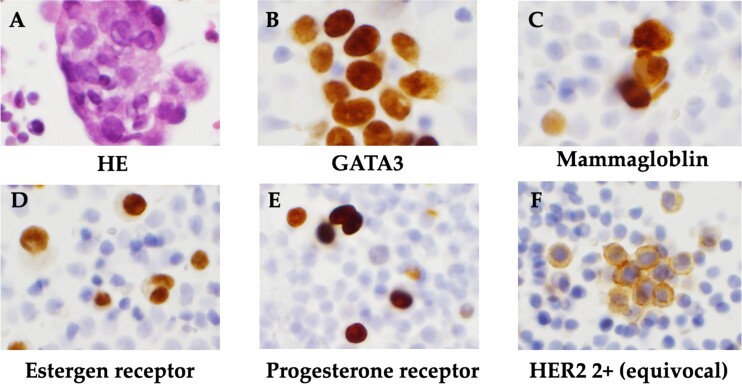
Histopathological findings. (A) HE staining showed a high N/C ratio, nuclear enlargement, and the appearance of solitary, scattered to small clusters of malignant cells of distinct glandular origin, suggesting an adenocarcinoma origin. (B) Cell block was positive for GATA3. (C) Cell block was positive for mammaglobin. (D) Positive immunohistochemistry for estrogen receptor. (E) Positive immunohistochemistry for progesterone receptor. (F) Immunohistochemistry showed HER2 was 2+ and no amplification by FISH.

The patient was a 59-year-old female. She had undergone total mastectomy and axillary lymph node dissection for right breast cancer 23 years previously, and had undergone postoperative hormone therapy for 2 years. Computed tomography (CT) scan of the abdomen taken at the time of initial examination revealed significant ascites accumulation and some intestinal obstruction-like findings (Fig. 1). An ascites puncture was performed, and cytological examination of the ascites fluid showed a high N/C ratio and a nucleus that was adenocarcinoma in origin. The cell block was positive for BerEP4, CK7, GATA3 and mammaglobin; weakly positive for E-cadherin; and negative for CK20 and PAX8. IHC biomarker showed estrogen receptor (ER) (+), progesterone receptor (PR) (−) and HER2 (2+) (no amplification by FISH), and the diagnosis of luminal (non-HER2) type was made (Fig. 2). Based on these results, the patient was diagnosed with peritoneal metastasis from breast cancer.

**Figure 3:**
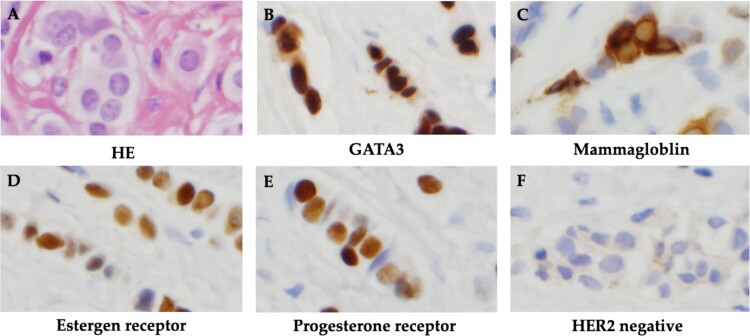
Histopathological findings. (A) HE staining showed atypical cells with rounded, slightly irregularly sized nuclei and chromatin-delicate nuclei with linear infiltration and proliferation. (B) Cell block was positive for GATA3. (C) Cell block was positive for mammaglobin. (D) Positive immunohistochemistry for estrogen receptor. (E) Positive immunohistochemistry for progesterone receptor. (F) Negative immunohistochemistry for HER2.

The patient was discharged after tamoxifen treatment resolved the ascites, and the symptoms of intestinal obstruction became less severe. Eleven months after the initial diagnosis, a subcutaneous mass appeared around the wound and surgical resection was performed for biopsy. Histopathological examination of the subcutaneous mass revealed linear infiltration and proliferation of atypical cells with round, slightly irregularly sized, chromatin-dense nuclei in the subcutaneous tissue. Cell blocks were positive for GATA3 and mammaglobin expression. IHC biomarker revealed ER(+), PgR(+) and HER2(−), and the metastatic lesion was diagnosed as a luminal (non-HER2)-type of breast cancer origin (Fig. 3).

**Figure 4:**
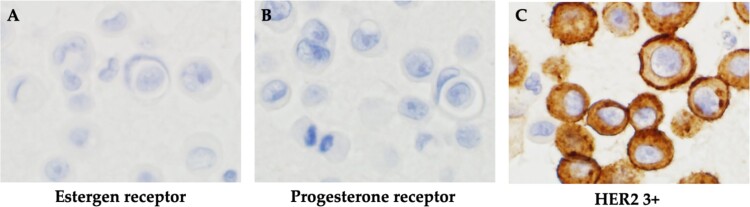
Histopathological findings. (A) Negative immunohistochemistry for estrogen receptor. (B) Negative immunohistochemistry for progesterone receptor. (C) Positive immunohistochemistry for HER2.

**Figure 5:**
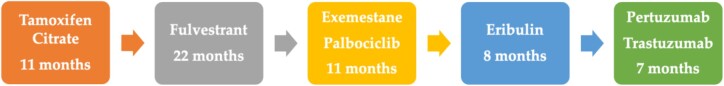
The course of treatment to date is illustrated.

The patient was switched from tamoxifen to fulvestrant, and control of the subcutaneous masses, and ascites was up to the expected level. After 22 months of a partial response to fulvestrant, bloating reappeared. After close examination, she was diagnosed with thoracoabdominal effusion and liver metastasis and was diagnosed to have progressive disease. The patient was treated with an aromatase inhibitor and a CDK4/6 inhibitor for 11 months, and the condition was stabilized for 11 months. Eight months after eribulin administration, the patient developed pleural effusion and the liver metastasis worsened. Cytological examination of pleural and ascites fluid suggested cancer metastasis. In the cell block, atypical cells with enlarged nuclei and distinct nucleoli appeared as small solitary clusters, and were positive for BerEP4, MOC31, GATA3, calretinin and D2–40. Based on these findings, breast cancer was diagnosed. In contrast, IHC biomarkers showed ER(−), PgR(−) and HER2 (3+) in both pleural and ascites fluids, and a diagnosis of the HER2 type (nonluminal) was made (Fig. 4). Therefore, the patient was offered anticancer drug and anti-HER2 therapy; however, she did not wish to take anticancer drugs, and treatment with pertuzumab and trastuzumab was initiated (Fig. 5). Seven months later, CT scans showed that the size of the thorax and abdomen had decreased, but the liver metastases had worsened. Multiple brain metastases were observed. The patient refused further treatment and died 2 months later.

## Discussion

Approximately 25% of patients with breast cancer develop distant metastasis, which can occur in different organs, such as the bone, lung, liver or brain. The 5-year survival rate is less than 20%, depending on the report, and the 5-year survival rate for liver metastasis is estimated to be less than 10% [2]. Recently, treatment options for metastatic breast cancer have been investigated based on BRCA gene testing, PD-L1 testing for triple-negative breast cancer and PIK3CA mutation status for ER-positive luminal breast cancer [3].

Immunohistochemistry is useful for the diagnosis of breast cancer metastases when the primary tumor is unknown. In this case, mammaglobin and GATA-binding protein 3 (GATA3) were positively expressed, and the immunohistochemistry of the metastatic lesion determined it to be a metastatic lesion originating from breast cancer. Mammaglobin and GATA3 play important roles in the identification of primary breast cancer metastatic sites. However, the details of various subtypes of breast cancer remain unknown [4]. GATA3 in cytological specimens is predominantly sensitive compared to GCDFP-15 and mammaglobin, and appears to be a marker specific for primary breast cancer compared to metastatic carcinomas of gynecological origin [5]. Therefore, GATA3 is useful for identifying breast origin and excluding gynecological origin in metastatic carcinomas of unknown primary origin.

These results suggest that aggressive re-biopsy may be useful to confirm changes in the biological characteristics of breast cancer metastases. Aggressive re-biopsy has been recommended in the past because the pathological diagnosis of advanced breast cancer metastases by re-biopsy is diverse and the possibility of puncture complications is low [6]. In metastatic breast cancer, the reported concordance rates of hormone receptors (ER and PR) and HER2 between primary tumors and metastases vary, and discordance is often observed. Discrepancies have been attributed to changes in key characteristics, heterogeneity within tumors, and sampling and analysis errors [7]. Therefore, it is recommended to re-search for hormone receptors and HER2 in metastases, if possible. Loss of hormone receptor expression has also been reported to be associated with decreased survival, and positive hormone receptor and HER2 expression may increase patient treatment options [3]. In this case, the hormone receptor and HER2 protein levels were completely reversed between the primary tumor and metastases, and the patient was able to use anti-HER2 drug therapy following HER2 positivity, resulting in long-term survival of more than six months. Although the patient did not want further treatment at the time of liver metastasis progression, anti-HER2 agents such as trastuzumab deruxtecan and trastuzumab emtansine could have resulted in further long-term survival.

## Conclusion

In conclusion, this case suggests that the cell block diagnostic method based on cytological diagnosis of pleural ascites fluid is useful not only for confirming the primary tumor but also for diagnosing the biological characteristics of breast cancer. In addition, we suggest that aggressive re-biopsy may be useful to confirm changes in the biological characteristics of breast cancer metastases.

## Data Availability

Data sharing is not applicable to this study because no datasets were generated or analyzed.
